# **­­**The emerging role of artificial intelligence enabled electrocardiograms in healthcare

**DOI:** 10.1136/bmjmed-2022-000193

**Published:** 2023-07-31

**Authors:** Arunashis Sau, Fu Siong Ng

**Affiliations:** 1 National Heart and Lung Institute, Imperial College London, London, UK; 2 Department of Cardiology, Imperial College Healthcare NHS Trust, London, UK; 3 Department of Cardiology, Chelsea and Westminster Hospital NHS Foundation Trust, London, UK

**Keywords:** Cardiology

**Key messages**

- Artificial intelligence has the potential to completely change the way physicians use electrocardiograms
- Applications of electrocardiograms enabled by artificial intelligence could include electrocardiogram interpretation, screening for prevalent disease, prediction of future disease, and phenotyping disease
- Advancements should be made with caution because of several potential pitfalls with the rapid growth of artificial intelligence enabled electrocardiogram applications

Artificial intelligence has the potential to completely change the way that physicians use the electrocardiogram, but caution must be applied, explain Sau and Ng

## Artificial intelligence, machine learning, and deep learning

Artificial intelligence (AI) as a field has had exponential growth and interest in the past 10 years. Clinicians have been promised previously unheralded insights and predictive power at their fingertips. Here, we discuss the basic principles of AI, how this applies to the electrocardiogram (ECG), and potential pitfalls with the rapid growth of AI in healthcare.

AI has been used in electrocardiograph machines for many years to provide computer interpretation of the ECG findings. However, the outcomes are often inaccurate and generally have little clinical use. The development of AI has led to a vast array of techniques that could completely change how we use the ECG.

A detailed overview of these topics has been covered in depth elsewhere.^1 2^ Briefly, AI is a branch of computer science dealing with machines performing tasks that usually would require human intelligence. Machine learning is a subbranch of AI and deals with the ability of machines to solve problems using inferences from data, rather than explicitly being programmed to accomplish a specific task.

## Supervised versus unsupervised machine learning

Supervised machine learning is by far the most common form of machine learning. This form involves provision of labelled data and allowing the model to identify important features that relate the input data to the label. For example, an ECG could be labelled with ECG abnormalities, such as left bundle branch block, or with a cardiac diagnosis, such as left ventricular dysfunction, and the algorithm can learn to classify ECGs based on these labels. Conversely, unsupervised learning does not use labelled data. Instead, the goal of this type of learning is to identify common features in the input data that could be used to cluster data into similar groups. In the healthcare setting, unsupervised learning would most commonly be used to cluster patients into similar phenogroups that may have different clinical features or outcomes.

## Traditional machine learning versus deep learning

**Figure 1:**
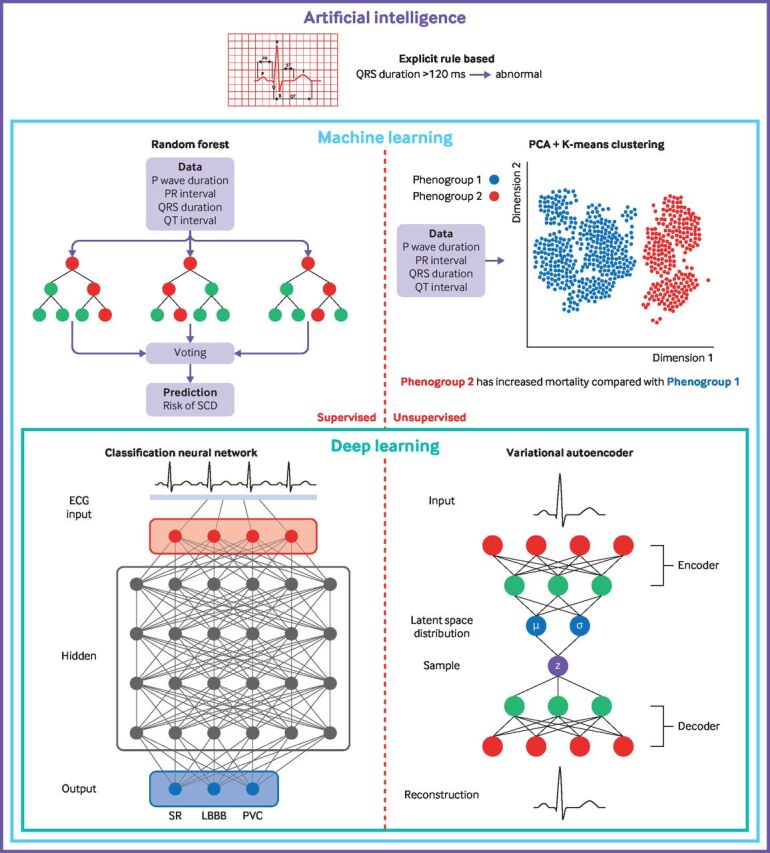
Examples of how artificial intelligence, machine learning, and deep learning can be applied to the ECG. Random forest is an ensemble of several uncorrelated decision trees, each using a random subset of features. Each tree makes a classification prediction, the class with the most trees with that prediction is the model’s final prediction. Principle component analysis (PCA) is a form of linear dimensionality reduction that can be applied, for example, to ECG measurements. PCA can be combined with clustering algorithms, such as K-means, to find clusters of patients who may have difference clinical features and outcomes. Neural networks are multilayered non-linear models that can be used to make connections within the input data and determine complex associations between input and label to make classification decisions. A variational autoencoder is a form of unsupervised deep learning where the network learns to compress the input into a latent space distribution before reconstructing and aiming to return to the same signal as the input. Variational autoencoders are often used for non-linear dimensionality reduction or removal of noise from signals. A single layer autoencoder with linear activation functions is almost equivalent to PCA. SCD=sudden cardiac death; SR=sinus rhythm; LBBB=left bundle block; PVC=premature ventricular contraction

Traditional machine learning involves human input for feature engineering. For example, a human might determine QRS duration to be an important feature for predicting left ventricular impairment. After feature engineering, various algorithms (including random forests as depicted in figure 1) can be used to learn from prespecified features and to then make classification or regression decisions.

**Figure 2:**
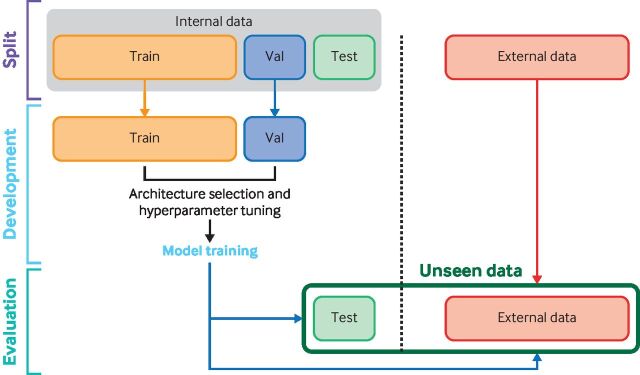
Flow of data in a machine learning study. An internal dataset is typically split into three sets: training (Train), validation (Val), and testing (Test). Training and validation sets are used for model development before evaluation on the unseen internal test set. An external dataset is ideally used in addition to the internal test dataset to ensure that the model generalises well to a different population

Conversely, deep learning takes the entire input (eg, the whole 12 lead ECG as a time series) without any human input for feature engineering and derives unique features from the data. This process has important advantages in that features that may not be apparent to humans can be used by the algorithm. Deep learning architectures are built from models called neural networks. Neural networks are structured with multiple layers, most of which are hidden layers (that is, between in the input and output layers) that are used to make connections within the input data and determine complex relations between input and label. Figure 1 depicts examples of how AI, machine learning, and deep learning can be applied to ECGs. Figure 2 shows how data should be used in the development and evaluation of a machine learning model.

## Advancing AI enabled ECGs

### Deep learning to improve ECG interpretation

Traditional computerised ECG interpretation uses a set of criteria developed by humans to make classification decisions based on the ECG input. These criteria are generally created from reference ranges in a population. For example, the computer interpretation on an ECG machine can be programmed to measure PR intervals, and where the PR interval is greater than 200 ms, the computer will make the diagnosis of first degree heart block. By contrast, the deep learning approach is able to identify important features from thousands of input ECGs and develop its own set of complex criteria for each diagnosis of interest. This more complex approach can be thought of as being analogous to how a clinician might develop skills in ECG reading over a career of ECG interpretation and clinical correlation. A deep learning approach to ECG interpretation could be more accurate than a traditional computerised interpretation,^3^ because ECG interpretations by deep neural networks might outperform skilled humans.^4^

### Screening for prevalent disease

Given the relative ease and low cost of performing an ECG, an important potential evolution in the application of AI enabled ECG is detection of prevalent disease. This distinction to the application of interpretation above is because AI enabled ECGs may be able to detect pathology that standard ECG interpretation by a clinician may miss. A prominent example is the impressive performance of AI enabled ECGs for the identification of asymptomatic left ventricular dysfunction (accuracy 85.7%, area under the receiver operating characteristics curve 0.93).^5^ AI enabled ECGs have also been applied for aortic stenosis detection^6^ (0.85). The ubiquitous ECG also provides an opportunity for screening for rarer diseases that may otherwise go undetected in current clinical practice. Hypertrophic cardiomyopathy by AI enabled ECG detection has achieved an impressive performance^7^(sensitivity 87%, specificity 90%, 0.96). AI enabled ECGs have also been applied for amyloidosis (0.91)^8^ and peripartum cardiomyopathy detection^9^ (area under the receiver operating characteristics curve of 0.87-0.92 depending on ejection fraction cut-off). These deep neural networks are able to identify both features that are apparent to humans and novel features that had not previously been described.^10^ Application of AI enabled ECGs for screening could be particularly relevant in primary care, where diseases currently may go undetected and could trigger referral on for further testing. However, we note several potential limitations. Any screening approach would need to be applied to appropriate populations, for example, opportunistic screening for asymptomatic left ventricular dysfunction in the general population may be appropriate given the high prevalence of the condition and the highly effective treatments that are available. By contrast, screening for rarer diseases without well established treatments, such as amyloidosis, is less likely to be beneficial. The potential harms of screening (including overdiagnosis and false positive diagnoses generating unnecessary additional testing) must be carefully considered and this approach needs evaluation in prospective clinical trials. Additionally, AI enabled ECG interpretation must be evaluated in an appropriate clinical context. For example, AI enabled ECGs may report evidence of aortic stenosis, but this finding may be incidental and not the cause of the patient’s symptoms.

### Prediction of future disease

The ECG is generally used by clinicians to identify prevalent abnormalities. AI enabled ECGs, however, have the potential to identify patients at risk of future disease. A potentially impactful application is the prediction of future atrial fibrillation from the sinus rhythm ECG (30 day prediction,^11^ area under the receiver operating characteristics curve 0.87; five year prediction, 0.909).^12^ This prediction could guide targeted screening, such as prolonged rhythm monitoring or mobile health devices. Another application of AI enabled ECG is to predict sudden cardiac arrest within 24 h^13^ (area under the receiver operating characteristics curve of 0.948 in an external dataset using 12 leads and 0.925 using a single lead I ECG). This screening might have the potential for use in a wearable device or implantable loop recorder in patients who are at an increased risk of cardiac arrest, but not sufficiently high risk enough to warrant implantable cardioverter defibrillator implantation. Although promising, further work is needed to evaluate potential applications of AI enabled ECG in this context.

### Disease phenotyping

Use of the ECG for unsupervised learning has potential to identify important disease phenotypes that may not be apparent from human inspection of clinical data and the ECG. Currently patients are selected for cardiac resynchronisation therapy comparatively crudely, by identification of left or right bundle branch block and measurement of QRS duration. Unsupervised machine learning has been applied to the raw QRS waveforms of patients who had cardiac resynchronisation therapy devices implanted for heart failure. Two prognostically important subgroups were identified, with importance beyond the crude parameters that we use today. The findings indicate unsupervised machine learning may be used to better identify patients who would benefit from cardiac resynchronisation treatment.^14^ Unsupervised machine learning, in the form of a convolutional autoencoder, has also been used to predict nocturnal hypoglycaemia.^15^ An autoencoder (depicted in figure 1) attempts to compress the input signal into a latent representation of features (in this case, 20 or 50 neurons) and then decompress to create a signal as close to the original as possible.^2^ Through this technique, the latent representation provides features that describe the input signal and can then be used for clustering or for a supervised machine learning task. The algorithm correctly identified clusters of ECGs based on glucose concentrations with 90% accuracy.

### Prospective validation of AI enabled ECG in clinical practice

Applications of AI enabled ECG have been extensively studied in retrospective datasets; however, prospective validation in clinical environments is important before clinical adoption. The first randomised clinical trial of AI enabled ECGs versus usual care was completed in 2021.^16^ They applied the AI enabled ECG algorithm for asymptomatic left ventricular dysfunction detection previously described^5^ in a cluster randomised trial of 120 primary care teams (22 641 patients). Overall, the AI enabled ECG group had a 32% increased rate of low ejection fraction diagnosis (odds ratio 1.32 (95% confidence interval 1.01 to 1.61), absolute diagnosis 2.1% *v* 1.6%) compared with usual care. AI enabled ECG could potentially be used in primary care to guide clinicians as to which patients should be referred for echocardiography. Importantly, echocardiogram use was not significantly different between the groups, indicating that AI enabled ECG may be a cost-effective tool, although formal cost effectiveness analysis is required.

**Figure 3:**
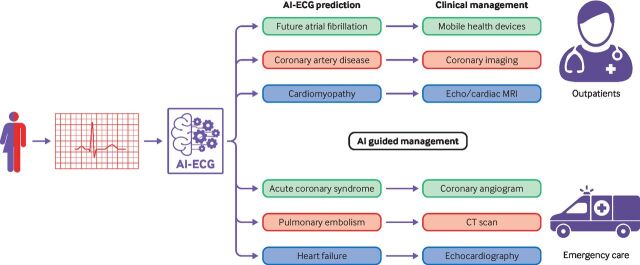
A depiction of how AI enabled ECG may be used in the near future to guide tailored further investigation and management in both outpatient and emergency settings. Examples of potential applications of AI enabled ECG prediction in each setting are given, with how these predications may guide clinical management

An observational study published in 2022 reported on the application of a deep neural network for left ventricular dysfunction screening embedded within an ECG enabled stethoscope.^17^ By use of an ECG enabled stethoscope, participants had a single lead ECG recorded, the area under the receiver operating characteristics curve was 0.85 for detecting left ventricular ejection fraction of less than 40%. Embedding AI enabled ECG within the familiar stethoscope may further increase its usability through quick recordings that can be taken in all patients who are seen in person. Figure 3 shows how AI enabled ECG may be used in the near future to support decision making for clinicians by providing suggestions for further investigation and management in both the outpatient and emergency settings. Despite the great potential of AI enabled ECG, improved clinical outcomes have not been shown; future studies will need to address this.

## Advancing with caution

Integration of AI into clinical workflows has the risk of important ethical issues. The World Health Organization has described six principles for the ethics and governance of artificial intelligence for health (Box 1),^18^ three of which we discuss.

Box 1 **World Health Organization principles for the ethics and governance of artificial intelligence for health**

- Protecting human autonomy
- Promoting human wellbeing and safety and the public interest
- Ensuring transparency, explainability, and intelligibility
- Fostering responsibility and accountability
- Ensuring inclusiveness and equity
- Promoting artificial intelligence that is responsive and sustainable

### Protecting human autonomy

The increasing role of AI in many fields, not just healthcare, risks the transfer of decision making power to machines. In healthcare, the risks are far too high, and many believe that humans must always have the final decision. The examples discussed in this review are predominantly about screening or identifying disease. In these scenarios, AI enabled ECG acts to enhance disease detection, where conditions could otherwise be missed, and in each case, a confirmatory test would generally be required before making any major changes to treatments. The potential exception to that is with respect to atrial fibrillation prediction. AI enabled ECG could be used to recommend pre-emptive anti-coagulation where the predicted risk of incident atrial fibrillation is particularly high. Any such workflows would need to be supported by clinical trials showing such a practice is beneficial and should always involve human input and shared decision making.

### Ensuring transparency, explainability, and intelligibility

One area of concern with deep learning models is the challenge in understanding reasons for the decision recommended by the model. Several methods, including saliency mapping, can partially overcome these limitations. A saliency map is an image where the brightness or colour of each pixel indicates how much of an impact that area of the image or signal has on predicting the outcome class. For example, a saliency map for a neural network designed to identify left bundle branch block would be expected to highlight the QRS of an ECG. However, no perfect method is available for explainability and all methods have important limitations.^19^ For example, saliency mapping is best suited to identifying incorrect foci for models rather than truly providing explainability. Using saliency mapping, a review of studies using AI models to detect covid-19 from a chest radiograph showed that many models used erroneous features.^20^ For example, parts of the image outside the lung fields such as the portable marker, which would generally reflect a more unwell patient, were used by the models to predict adverse outcomes.

Another thought is that interpretability is not required for adoption of AI models into clinical practice. In striving for explainability in our AI models, some people argue that we may limit machines to the level of reasoning that humans are capable of, and therefore deprive the world of the unique problem solving capabilities of AI.^21^ Furthermore, AI is held to higher standards than other tools used by clinicians. Several examples exist of medical treatments and procedures where the exact mechanism of benefit is not clearly understood but treatments are used extensively after favourable data was available from clinical trials.^22 23^ Human clinical decision might often escape explainability,^24^ so why should AI be held to this potentially unrealistic target? Human clinical decision making may also be influenced by unmeasured factors relating to human interaction, such as facial expressions that may convey anxiety or degree of pain. These factors would not be considered by most current AI models. The answer to this question is likely a compromise between these two extremes—some explainability in AI is necessary; however, requiring complete understanding of the AI model is probably an unrealistic goal that will stifle innovation if pursued rigorously.

### Ensuring inclusiveness and equity

AI has the potential to both widen and close existing forms of bias and discrimination. Examples from image analysis from large datasets have shown poor performance on images of people from ethnic minorities, due to the largely white population used for training the AI model.^25^ On the contrary, other studies have shown that AI enabled ECG may in fact perform equally well for people from ethnic minorities, even when they did not comprise a large proportion of the training cohort.^17 26^. Rigorous testing must be used to ensure systemic biases are not introduced through application of AI enabled ECG.

### The present situation

AI enabled ECG is not currently ready for clinical use, however, prospective clinical studies have shown much promise. Figure 3 depicts potential applications for AI enabled ECGs in both the outpatient and emergency settings. The earliest clinical applications of AI enabled ECGs are likely to be additive to current clinical pathways, for example, in screening for asymptomatic left ventricular dysfunction or for patients at high risk of occult atrial fibrillation. Yet, substantial work remains in order to fully realise the potential of AI enabled ECG.

## Conclusion

Deep learning has the potential to completely change the use of the ECGs and provide hidden mechanistic and clinical insights. The use of AI enabled ECG could transform clinical care of patients with cardiovascular disease, promoting early detection and tailored therapy. However, as with the implementation of all AI tools, great care must be taken to ensure the implementation of AI enabled ECG is done safely and ethically.

## Data Availability

No data are available.
